# Supplementary material for: The causal relationship between the human gut microbiota and pyogenic arthritis: a Mendelian randomization study
Source: Front Cell Infect Microbiol. 2024 Nov 26;14:1452480. doi: 10.3389/fcimb.2024.1452480 (PMC11629706; doi:10.3389/fcimb.2024.1452480)
Supplement: Supplementary file 1 [file DataSheet1.zip › mendelian test/ebi-a-GCST90027710.csv_leave_one_out.pdf]

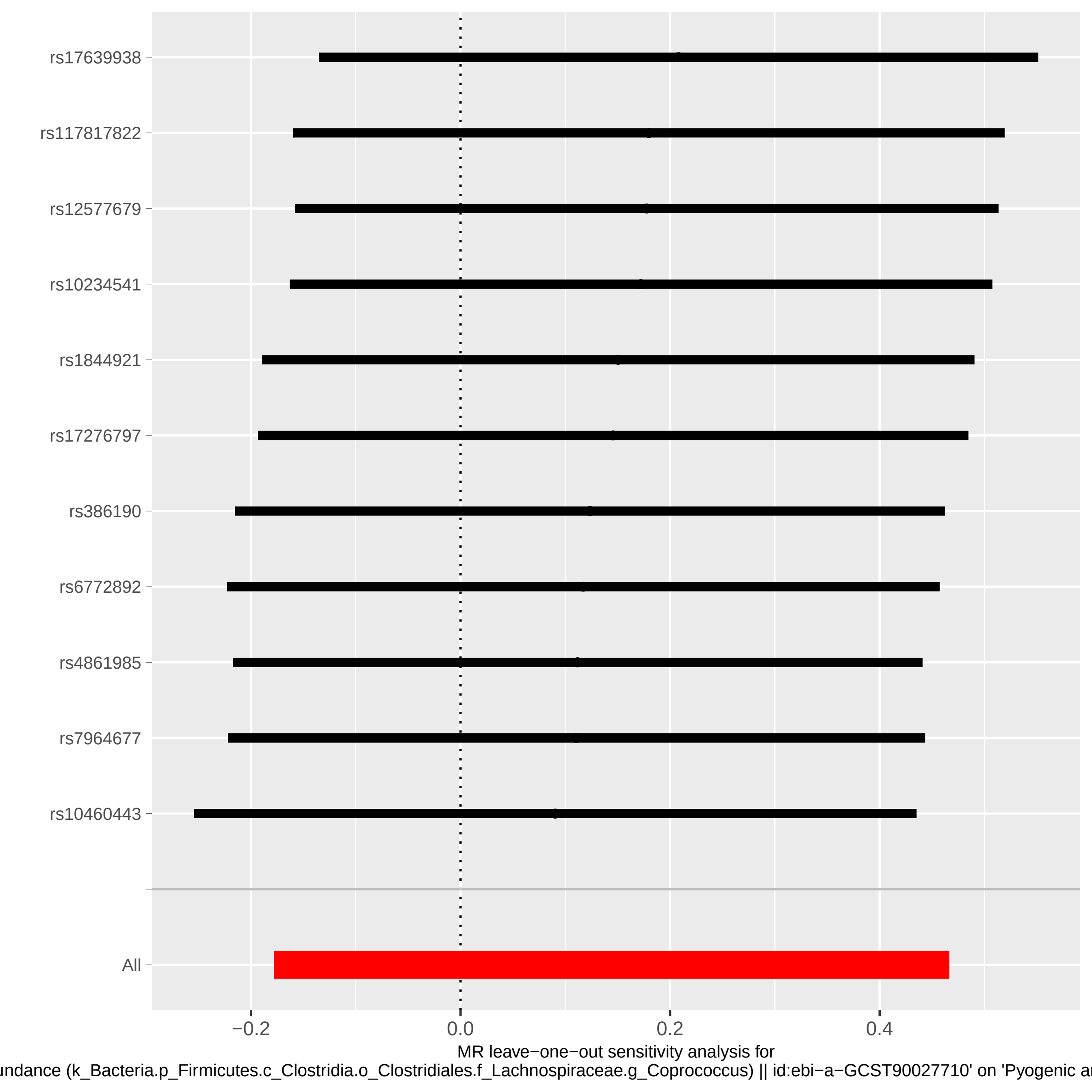
rs17639938
rs117817822
rs12577679
rs10234541
rs1844921
rs17276797
rs386190
rs6772892
rs4861985
rs7964677
rs10460443
All
−0.2
0.0
0.2
0.4
MR leave−one−out sensitivity analysis for
undance (k_Bacteria.p_Firmicutes.c_Clostridia.o_Clostridiales.f_Lachnospiraceae.g_Coprococcus) || id:ebi−a−GCST90027710' on 'Pyogenic ar
